# Moderating Effect of Grip Strength in the Association between Diabetes Mellitus and Depressive Symptomatology

**DOI:** 10.3390/sports12010003

**Published:** 2023-12-20

**Authors:** Diogo Veiga, Miguel Peralta, Élvio R. Gouveia, Laura Carvalho, Jorge Encantado, Pedro J. Teixeira, Adilson Marques

**Affiliations:** 1 CIPER, Faculdade de Motricidade Humana, Universidade de Lisboa, 1499-002 Cruz-Quebrada, Portugal; dmcveiga@fmh.ulisboa.pt (D.V.); laura.carvalho@e-fmh.ulisboa.pt (L.C.); jencantado@fmh.ulisboa.pt (J.E.); pteixeira@fmh.ulisboa.pt (P.J.T.); amarques@fmh.ulisboa.pt (A.M.); 2 ISAMB, Faculdade de Medicina, Universidade de Lisboa, 1649-026 Lisboa, Portugal; 3 Department of Physical Education and Sport, University of Madeira, 9020-105 Funchal, Portugal; erubiog@staff.uma.pt; 4 LARSYS, Interactive Technologies Institute, 9020-105 Funchal, Portugal

**Keywords:** depression, elderly, grip strength, moderation, diabetes

## Abstract

Diabetes mellitus and depression rank among the leading causes of disease burden and are present in the top ten causes of disability-adjusted life years worldwide. Numerous studies have shown that both depression and diabetes have a detrimental effect on the quality of life, and when they coexist, the effect is considerably worse. This study aimed to analyse how grip strength moderates the relationship between diabetes and depressive symptoms among middle-aged and older adults. In total, 41,701 participants (18,003 men) in wave 8 of the cross-sectional population-based Survey of Health, Ageing, and Retirement in Europe (2019/2020) data were studied. A dynamometer was used to test grip strength twice on each hand. Depressive symptoms were measured using the 12-item EURO-D scale. The relationship between diabetes and depressive symptoms is negatively moderated by grip strength (male: B = −0.03, 95% CI = −0.04, −0.03; female: B = −0.06, 95% CI = −0.07, −0.06). Furthermore, the significant zone grip strength moderation values for males and females were less than 48.7 kg and 38.9 kg, respectively. Muscular strength was a moderator of depressive symptoms, attenuating its association with diabetes. This supports the premise that physical activity, namely muscle-strengthening exercises, should be included in diabetes treatment programs.

## 1. Introduction

Chronic hyperglycemia is a hallmark of diabetes mellitus (DM), a metabolic disorder involving the metabolism of carbohydrates, proteins, and fats that has been extensively investigated [1]. There are two main types of diabetes. Type 1 DM is an autoimmune disease that leads to the destruction of insulin-producing pancreatic beta cells [2]. The two main causes of type 2 DM are the incapacity of insulin-sensitive tissues to react to insulin correctly and impaired insulin production by pancreatic β-cells [3]. Over the next ten years, the prevalence of DM is anticipated to rise by 25% globally, making it a rising public health concern [4].

Depression is a significant comorbid condition in DM [5]. According to the Diagnostic and Statistical Manual of Mental Disorders, Fifth Edition (DSM-5), the diagnosis of depression requires five or more symptoms to be present within a 2-week period [6]. One of the symptoms should, at least, be either a depressed mood or anhedonia. The secondary symptoms are appetite or weight changes, sleep difficulties, psychomotor agitation or retardation, fatigue or loss of energy, a diminished ability to think or concentrate, feelings of worthlessness or excessive guilt, and suicidality. A key contributor to disability, depression lowers the quality of life, raises the chance of early death, and heavily strains healthcare systems [7]. Different estimates show that depression could affect more than 300 million people worldwide [8,9].

DM and depression rank among the leading causes of disease burden worldwide and are present in the top ten causes of disability-adjusted life years worldwide [10]. Evidence supports a bidirectional association between DM and depression [11]. DM increases the risk of depression by 15–28% [12]. On the other hand, depression was confirmed as a risk factor for DM (34% and 60% risk increase) [13]. Numerous studies have shown that both depression and DM have a detrimental effect on the quality of life, and when they coexist, the effect is considerably worse [14,15,16,17]. Even when other known DM risk factors are considered, such as a poor diet [18], family history [19], inflammation [19], the use of some antidepressants [20], and a sedentary lifestyle [21], the elevated risk for DM associated with depression persists [22].

Physical activity, defined as any bodily movement produced by skeletal muscles that results in energy expenditure [23], provides various benefits, including preventing chronic diseases, lowering the risk of depression, increasing physical strength, and reducing mental stress [24]. A person’s capacity to do physical activity is correlated with a set of traits known as physical fitness [25]. The health-related components of physical fitness are cardiorespiratory endurance, muscular endurance, muscular strength, body composition, and flexibility. From all the health-related components of physical fitness [23], muscular strength has been researched recently regarding its relationship with middle-aged and older adults’ mental and physical health [26]. Among people with DM, strength training may not only help alleviate depressive symptoms [27], but also assist in achieving glycaemic control [28]. Regular physical activity helps regulate normal glucose uptake into peripheral tissues, increases insulin receptors, and improves insulin sensitivity [29], thus contributing to the blood glucose control [30]. Strength training has anti-inflammatory properties [31], aids in beneficial changes in body composition [32], and makes weight management easier [33]. In addition, muscle strength seems to be inversely correlated with depression and cognitive performance in middle-aged and older adults [34,35], emphasising the value of exercise and physical fitness in preventing depression. Grip strength (GS), the force used by the hand and fingers to grasp and hold onto an object, is a numerical assessment of the strength of the muscles used in handgrip activities [36]. GS, a measure of muscle strength that is associated with physical activity levels [37], has been considered a legitimate and trustworthy measure of overall muscle strength and the state of one’s physical and mental health [38].

Given that the prevalence of both DM and depression is increasing in the middle-aged and older adults’ group [39,40], and the causes and relationship remain rather complex and understudied [41], this study aimed to analyse how GS moderates the relationship between DM and depressive symptoms among middle-aged and older adults.

## 2. Materials and Methods

### 2.1. Participants and Procedures

Data from the Survey of Health, Ageing, and Retirement in Europe (SHARE) wave 8 data (2019/2020) were used. The SHARE methodology has been previously described [42]. This survey gathers data on adults aged 50 and above every two years in European countries and Israel. The target population consists of all people living in residential households, plus their (possibly younger) partners. Those who do not reside at the sampled address (e.g., because it was a seasonal or vacation residence), are physically or mentally unable to participate, have died before the start of the field period, or cannot speak the specific language of the national questionnaire were excluded. The SHARE protocol was approved by the University of Mannheim Ethics Committee and the Ethics Council of the Max-Planck-Society for the Advancement of Science. The study’s participants all provided their written informed consent.

There were 41,701 participants (30,224 with age ≥ 65), 18,003 men and 23,698 women, with a mean age of 70.65 (9.1) and coming from 29 different countries (Austria, Germany, Sweden, The Netherlands, Spain, Italy, France, Denmark, Greece, Switzerland, Belgium, Israel, the Czech Republic, Poland, Ireland, Luxembourg, Hungary, Portugal, Slovenia, Estonia, Croatia, Lithuania, Bulgaria, Cyprus, Finland, Latvia, Malta, Romania, and Slovakia).

### 2.2. Measures

The outcome measure was depressive symptoms. The EURO-D 12-item scale was used to quantify depressive symptoms. Scores range from 0 to 12, with higher numbers denoting more severe depressive symptoms. A cut-off ≥ 4 points indicates clinically significant depression [43,44]. The scale’s validation and justification are discussed elsewhere [44]. The exposure measure was DM. The existence or absence of DM that had previously been diagnosed by a doctor was inquired of the participants. GS was the moderator in use. It was measured twice on each hand using a dynamometer (Smedley, S Dynamometer, TTM, Tokyo, 100 kg), switching between the left and right hands [45]. The inner lever of the dynamometer was set to the hand. Participants kept their upper arm tightly against their bodies while standing or sitting, with the elbow at a 90-degree angle, the wrist in neutral, and the elbow snugly against their body. Participants exerted the dynamometer’s maximum pressure for 5 s. Before the assessment, participants had the option to practice. The GS variable contained the maximum value of the GS measurement of both hands. Values of two measures that differed by more than 20 kg were considered invalid. Measurements of GS that were equal to or more than 100 kg were excluded, as well as measurements where GS was only assessed once in one hand. Sex and age were the self-reported covariates.

### 2.3. Statistical Analysis

**Figure 1:**
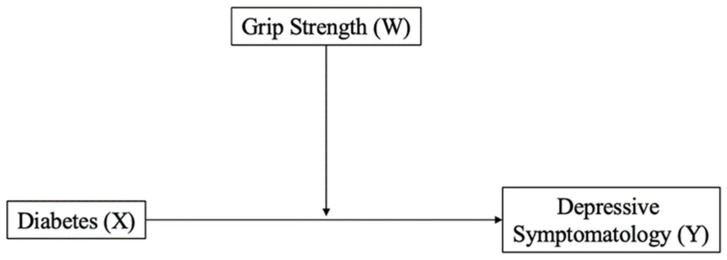
A conceptual diagram of the relationship between diabetes (X) and depressive symptomatology (Y) moderated by grip strength (W).

Descriptive statistics (mean, standard deviation, and frequency) were calculated for all variables. The depressive symptomatology of men and women according to DM diagnosis, as well as the association between GS and depressive symptomatology, were compared using an independent sample *t*-test and a Pearson correlation analysis, respectively. Using the suggestions for moderation made by Baron and Kenny [46], a moderated analysis of GS (moderator, W) on the connection between DM (categorical, X) and depressive symptoms (continuous, Y) was conducted (Figure 1). The moderation analysis was carried out using Andrew Hayes’ PROCESS macro-3.5. The Johnson-Neyman method was used to evaluate statistically significant interactions and find regions of significance. This process was also used to determine a threshold of statistical significance. Analysis was stratified by sex and adjusted for age. Data analysis was performed using IBM SPSS Statistics version 28 (SPSS Inc., an IBM Company, Chicago, IL, USA). For all tests, the statistical significance was set at *p* < 0.05.

## 3. Results

The descriptive analysis is presented in Table 1. Women (45.6%) reported having depressive symptoms more frequently than men (29.6%). Men, however, were more likely than women to report having been diagnosed with DM (16.2% vs. 12.7%).

Table 2 presents the relationship between GS and depressive symptoms. GS was negatively correlated with depressive symptoms for the entire sample (r = −0.254, *p* < 0.001) as well as for males and females separately (r = −0.193, *p* < 0.001 vs. r = −0.210, *p* < 0.001).

The depressive symptoms of patients with and without DM are presented in Table 3. The mean depressive symptomatology was higher in people with DM than those without DM, regardless of sex (males: 2.53 vs. 1.76, *p* < 0.001; females: 3.55 vs. 2.50, *p* < 0.001).

The moderating role of GS (W) in the relationship between DM (X) and depressive symptoms (Y) is shown in Table 4. (male: B = −0.03, 95% CI = −0.04, −0.03; female: B = −0.06, 95% CI = −0.07, −0.06) means that a higher GS resulted in a reduced correlation between DM and depressive symptoms. Additionally, the Johnson-Neyman test showed that the GS moderation values for males and females in the significant zone were less than 48.7 kg and 38.9 kg, respectively.

## 4. Discussion

This study investigated how GS affects the relationship between depressive symptomatology and DM in middle-aged and older individuals. According to the findings, depressive symptoms were significantly higher in both men and women with DM, and GS was significantly associated with lower depressive symptoms. When getting a DM diagnosis, GS reduced depressive symptoms, moderating the relationship between them and DM.

Depression is more common in women than in men. According to research, women are roughly twice as likely as men to experience depression throughout their lifetime [47]. This sex difference in depression prevalence aligns with our results reporting that women suffer more from depression than men (45.6% and 29.6%, respectively). The reasons for this disparity are multifaceted and may involve a combination of biological, psychological, and sociocultural factors [48].

Our results have shown a negative correlation between GS and depressive symptoms, meaning that as GS increases, the severity of depression tends to decrease. Various studies also observe this association [49,50]. The exact mechanisms underlying this correlation have yet to be fully understood, but several potential explanations exist. For groups with co-occurring mental and physical health issues, and once it is often considered a proxy for overall physical health and functional fitness, GS might offer four advantages: (1) it might lessen depressive symptoms through biological and psychosocial mechanisms; (2) it might enhance physical health by treating the comorbid illness itself or by avoiding its secondary effects; (3) it may be that individuals with better physical health, including muscle strength, have a more robust physiological response to stress and are better equipped to cope with depressive symptoms; and (4) it might also reflect individuals who are involved in physical activities that confer mental health and well-being benefits [28].

The guidelines of scientific societies indicate that a substantial proportion of patients are diagnosed with a psychiatric disorder [51]. Given the chronic nature of DM, along with the daily management tasks and potential complications, people with DM have a higher risk of developing depression compared to the general population [52]. The constant need for self-management can be stressful and overwhelming, contributing to frustration and anxiety. Our research reveals that in men and women, participants with DM tended to present higher scores of depressive symptomatology than those without it. This increased risk can be attributed to various factors related to living with DM. Through the dysregulation of the hypothalamic-pituitary-adrenal axis, the hyperactivity of the autonomic nervous system, and inflammatory processes, depression and type 2 DM share biological roots. Type 1 DM patients may be more vulnerable to depression if they have a long-lasting disease from a young age when their personalities are also forming [10].

A moderator is a variable that can influence or modify the relationship between two other variables: in this case, DM and depressive symptomatology. Our findings demonstrate that for both males and females, GS modifies the link between depressive symptomatology and DM. B’s negative value reveals that the association is unfavourable. This indicates that rather than negating the link between these two variables, the moderating effect weakens it. According to these findings, those who have DM typically experience more depression than those who do not (higher score values for depressive symptoms). Nevertheless, among those who do, individuals with greater muscular strength, as determined by GS, tend to experience less suffering (show lower scores of depressive symptoms) than those with lesser muscular strength. According to the Johnson-Neyman test, this moderation effect is present for GS values below 48.7 kg for men and 38.9 kg for women.

Several things could justify that individuals with more muscular strength may be less susceptible to the detrimental psychological effects of DM. A physically active lifestyle and improved mental health frequently correlate with a stronger GS [53]. Based on preliminary data, resistance training appears to be beneficial for enhancing the majority of life quality domains, handgrip strength, lower and upper limb muscle strength, and depression in middle-aged and older adults [54]. Regular exercise, including strength training, can lessen both stress and the symptoms of depression. In parallel, strength exercises can boost self-efficacy and self-confidence while helping to improve GS [55]. These psychological elements may provide protection from the emotional strain that comes with managing DM. Also, exercise and strength training, in general, are efficient stress-reduction methods [56]. Regular strength training may help people with DM to cope better with the pressures of the condition, thus lowering the risk of depression.

The moderating impact of GS on depressive symptomatology may not be the same for every DM patient. It is conceivable that the degree to which GS moderates the association between DM and depression varies depending on things like: DM severity (the moderating effect may be more prominent in people with poorly controlled DM or those who have had the condition for a longer period), individual resilience (the effect of DM on depression may depend on an individual’s psychological resilience, coping mechanisms, and social support system), and ageing (GS usually weakens with age and younger people with DM may experience a different moderating impact than middle-aged and older persons).

The strengths and limitations of the existing results should be considered when analysing them. Regarding the limitations, the study’s cross-sectional design cannot prove causality because it is mainly descriptive. The direction of causality or the existence of underlying causal mechanisms cannot be determined. However, they can spot relationships or correlations between variables. Another limitation was the inability to account for several confounding factors, such as smoking use/abuse, educational level, diet, or physical activity levels. Regarding the strengths, this is the first investigation, as far as we are aware, on the moderating effect of GS in the association between depressive symptoms and DM. In this study, physical fitness was measured by an objective measure (i.e., GS) acting as a moderator and assessed by an impartial physical fitness test. Objective measures provide precise and accurate data, reducing the potential for measurement error or bias. They offer clear and quantifiable results that can be consistently obtained across different assessments and evaluators [57]. It also constitutes a strength that this study was with a multinational sample. Multinational samples, which capture a wide range of cultural, social, and economic viewpoints, produce conclusions more generally applicable to a global environment. Because they are less likely to be impacted by peculiar or regional characteristics, the results from international samples are frequently more solid and reliable. The validity of results is strengthened by consistency across various populations [58].

Research into the relationship between muscular strength and depression in people with DM is underway. This study highlights the possibility that, even when diagnosed with DM, persons with higher grip strength values may have a mitigating effect on depressive symptoms. There is already evidence that suggests that GS may lessen depression in people with DM, but further studies are required to fully understand the processes at work and to pinpoint the precise circumstances in which this moderation takes place. This link is complicated and not entirely clear. It is crucial to understand that DM patients’ despair is not solely influenced by their GS. Future research in this area should consider that other elements, like glycemic control, healthcare access, social support, and psychological elements, can also greatly impact how depression develops and is treated in this population. A specific focus should be placed on middle-aged and older individuals because DM and depression are becoming more prevalent in this age group. Due to the anticipated rise in the population’s senior citizens, who have a high prevalence of DM, this is especially significant.

## 5. Conclusions

Muscular strength moderated depressive symptoms, attenuating its association with DM and supporting the premise that physical activity, namely muscle-strengthening exercises, could be included in DM treatment programs. In addition to taking other comorbidities into account when managing DM, depression should also be considered. Priority should be given to the early detection of depressive symptoms in DM patients who are depressed to prevent the sad state from adversely affecting the clinical management of DM. Furthermore, because GS is a simple, quick, and economical test, DM recovery programs could include physical activity, namely muscle-strengthening exercises, as one strategy to potentially prevent depression.

## Figures and Tables

**Table 1:** Sample characteristics for the total sample and by sex.

	Mean (SD) or *n* (%)		
	Total (*n* = 41,701)	Male (*n* = 18,003)	Female (*n* = 23,698)
Age (years)	70.65 (9.1)	71.1 (8.8)	70.3 (9.4)
Grip strength (kg)	32.0 (11.2)	40.7 (10.0)	25.5 (6.7)
EURO-D score	2.3 (2.2)	1.9 (1.9)	2.6 (2.3)
Diabetes			
Yes *n* (%)	5932 (14.2)	2918 (16.2)	3014 (12.7)
No *n* (%)	35,769 (85.8)	15,085 (83.8)	20,684 (87.3)
Depression			
Yes *n* (%)	16,130 (38.7)	5331 (29.6)	10,799 (45.6)
No *n* (%)	25,571 (61.3)	12,672 70.4)	12,899 (54.4)

Abbreviations: SD, standard deviation.

**Table 2:** Pearson correlation between grip strength and depressive symptomatology.

	Depressive Symptoms (EURO-D 12 Score)					
	Total		Male		Female	
	r	*p*-Value	r	*p*-Value	r	*p*-Value
Grip strength	−0.254	<0.001	−0.193	<0.001	−0.210	<0.001

**Table 3:** Comparison of depressive symptomatology according to the presence or absence of diabetes.

	Depressive Symptoms (EURO-D 12 Score)					
	Total		Male		Female	
	Mean (SD)	*p*-Value	Mean (SD)	*p*-Value	Mean (SD)	*p*-Value
With diabetes	2.71 (2.32)	<0.001	2.18 (2.08)	<0.001	3.22 (2.43)	<0.001
Without diabetes	2.25 (2.12)		1.85 (1.92)		2.54 (2.21)	

**Table 4:** Moderation analysis of grip strength for the relationship between diabetes and depressive symptomatology stratified by sex.

	Depressive Symptoms (EURO-D 12 Score)					
	Total Sample		Male		Female	
	B	95% CI	B	95% CI	B	95% CI
Diabetes (X)	0.94	0.77, 1.11	0.67	0.35, 0.98	0.98	0.67, 1.29
Grip Strength (W)	−0.04	−0.05, −0.04	−0.03	−0.04, −0.03	−0.06	−0.07, −0.06
Diabetes*Grip Strength	−0.02	−0.02, −0.01	−0.01	−0.02, 0.00	−0.02	−0.03, −0.01

Abbreviations: CI, confidence interval.

## Data Availability

The data are freely accessible. The data can be accessed through the SHARE project website—https://share-eric.eu/ (accessed on 21 June 2023).
